# New insights from GWAS for the cleft palate among han Chinese population

**DOI:** 10.4317/medoral.21439

**Published:** 2017-02-04

**Authors:** Shi-Jun Duan, Ning Huang, Bi-He Zhang, Jia-Yu Shi, Sha He, Jian Ma, Qiong-Qiong Yu, Bing Shi, Zhong-Lin Jia

**Affiliations:** 1 PhD, State Key Laboratory of Oral Diseases, West China Hospital of Stomatology, Sichuan University, Chengdu, China; 2 MD, PhD, State Key Laboratory of Oral Diseases, West China Hospital of Stomatology, Sichuan University, Chengdu, China; 3 Msc, State Key Laboratory of Oral Diseases, West China Hospital of Stomatology, Sichuan University, Chengdu, China; 4 PhD, Division of Growth and Development and Section of Orthodontics, School of Dentistry, University of California, Los Angeles, USA

## Abstract

**Background:**

Genome wide association studies (GWAS) already have identified tens of susceptible loci for nonsyndromic cleft lip with or without cleft palate (NSCL/P). However, whether these loci associated with nonsyndromic cleft palate only (NSCPO) remains unknown.

**Material and Methods:**

In this study, we replicated 38 SNPs (Single nucleotide polymorphisms) which has the most significant *p* values in published GWASs, genotyping by using SNPscan among 144 NSCPO trios from Western Han Chinese. We performed the transmission disequilibrium test (TDT) on individual SNPs and gene-gene (GxG) interaction analyses on the family data; Parent-of-Origin effects were assessed by separately considering transmissions from heterozygous fathers versus heterozygous mothers to affected offspring.

**Results:**

Allelic TDT results showed that T allele at rs742071 (PAX7) (*p*=0.025, ORtransmission=3.00, 95%CI: 1.09-8.25) and G allele at rs2485893 (10kb 3’ of SYT14) were associated with NSCPO (*p*=0.0036, ORtransmission= 0.60, 95%CI: 0.42-0.85). Genotypic TDT based on 3 pseudo controls further confirmed that rs742071 (*p*-value=0.03, ORtransmission=3.00, 95%CI: 1.09-8.25) and rs2485893 were associated with NSCPO under additive model (*p*-value= 0.02, ORtransmission= 0.66, 95%CI: 0.47-0.92). Genotypic TDT for epistatic interactions showed that rs4844913 (37kb 3’ of DIEXF) interacted with rs11119388 (SYT14) (*p*-value=1.80E-08) and rs6072081 (53kb 3’ of MAFB) interacted with rs6102085 (33kb 3’ of MAFB) (*p*-value=3.60E-04) for NSCPO, suggesting they may act in the same pathway in the etiology of NSCPO.

**Conclusions:**

In this study, we found that rs742071 and rs2485893 were associated NSCPO from Han Chinese population; also, interactions of rs4844913:rs11119388 and rs6072081:rs6102085 for NSCPO were identified, gene-gene interactions have been proposed as a potential source of the remaining heritability, these findings provided new insights of the previous GWAS.

**Key words:**GWAS, NSCPO, TDT, parent-of-origin effects, epistatic interactions.

## Introduction

Cleft palate (CP) is a common birth defect, which has a lower birth prevalence compared to cleft lip with/without cleft palate (CL/P): 1/2500 live births vs. 1/700; but CP shows less variability in birth prevalence across populations compared to CL/P (1).

In nonsyndromic cleft palate only (NSCPO), affected individuals have no other physical or developmental anomalies. Most studies suggest that about 50% of CP is nonsyndromic (2). Both population studies and family studies suggested that genetic factors played a critical role in the etiology of NSCPO (3,4). Among first degree relatives, the relative risks of recurrence risks were 56 for cleft palate only and 32 for any cleft lip when compared to the general population in Norway (3); several genes have been identified for syndromic forms of CP, few have been identified as influencing risk to NSCPO. The etiology of this complex trait has been widely studied in order to search for the risk factors and to design strategies for prevention.

Genome wide association studies entail the matching of a given human genome sequence with an annotated, high-resolution map of common genetic variation; They are contributing a lot to our understanding of diseases to which there is a genetic predisposition (5). Genome wide association studies (GWAS) already have identified tens of susceptible loci for cleft lip with or without cleft palate (CL/P).

However, whether these loci associated with nonsyndromic cleft palate only for Han Chinese remains unknown. In this study, we replicated 38 SNPs from 19 genes/ regions of 11 chromosomes from previous GWAS (6-10) and other studies (11-13) with prior compelling evidence contributing to NSCL/P to investigate their roles in Han Chinese population.

## Material and Methods

- Samples

Our samples consisted of 144 complete case-parent trios with nonsydromic cleft palate only (NSCPO), 59 males, 82 females and 3 unknown gender of the probands. All subjects were self-identified as Western Han Chinese, they were recruited between 2008 and 2013 from the Cleft Surgery Department of West China Hospital of Stomatology, Sichuan University. Written informed consent was obtained from parents on behalf of the children and all affected individuals old enough to give their own consent in this study. The consent procedure and this study approved by the Hospital Ethics Committee (HEC) of West China Hospital of Stomatology, Sichuan University.

- Genotyping

Venous blood samples were drawn from participants and DNA was extracted by phenol chloroform extraction protocol. The SNP genotyping work was performed using a custom-by-design 2x48-Plex SNPscanTM Kit (Cat#:G0104, Genesky Biotechnologies Inc., Shanghai, China).

- Statistical analysis 

The unaffected parents were underwent Hardy-Weinberg Equilibrium (HWE) analysis and minor allele frequency (MAF) determination. HWE, MAF, allelic TDT and parent-of-origin effects, were calculated using PLINK (14). Pairwise LD as both their D’ and r2 were computed for all the SNPs using the haploview program (http://www.broad.mit.edu/haploview/haploview). Genotypic TDT and Likelihood ratio test for epistatic interactions based on genotypic TDTs were determined with R Package trio (v1.4.23) (15), all two-way interactions comprised a matrix in genotype format were tested using the function colGxG, without specifying the genes, and the interactions between all 38 (38-1)/2 pairs of the SNPs in a matrix were tested. We used a Bonferonni correction for 38 tests to determine a threshold for formal significance of *p*=0.0013.

## Results

- Transmission Disequilibrium Test

All SNPs were passed the HWE test (*p* >0.05) ( Table 1). Allelic TDT results showed that T allele at rs742071 (PAX7) (*p*=0.025, ORtransmission=3.0, 95%CI: 1.09-8.25) and G allele at rs2485893 (10kb 3’ of SYT14) were associated with NSCPO (*p*= 0.0036, ORtransmission= 0.60, 95%CI: 0.42-0.85) ( Table 2). Genotypic TDT based on 3 pseudo controls further confirmed that rs742071 (*p*-value=0.03, ORtransmission=3.0, 95%CI: 1.09-8.25) and rs2485893 are associated with NSCPO under additive model (*p*-value=0.02, ORtransmission=0.66, 95%CI: 0.47-0.92) ( Table 3).

**Table 1:**
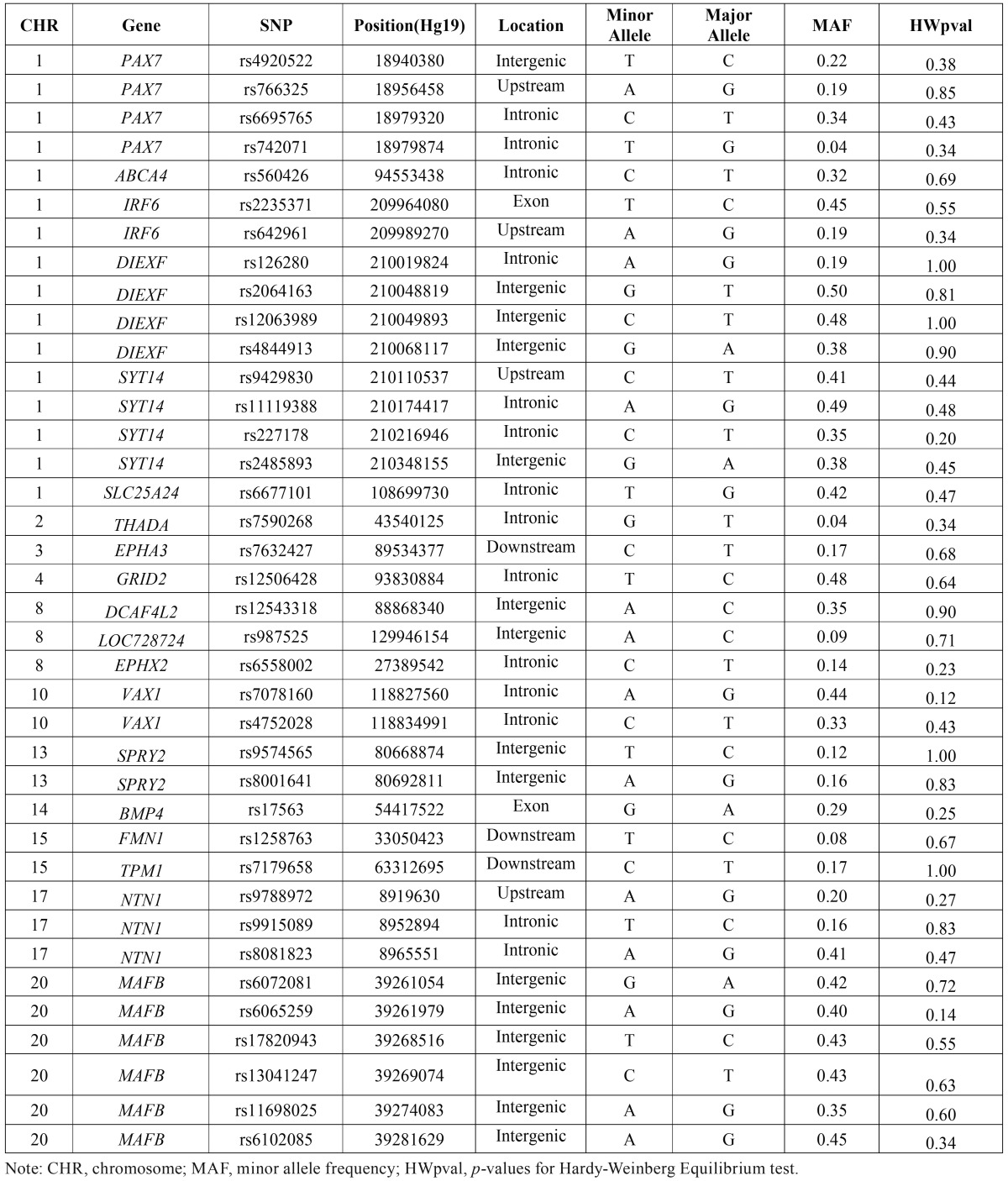
Minor allele frequency and Hardy-Weinberg Equilibrium Test of the SNPs for NSCPO.

**Table 2:**
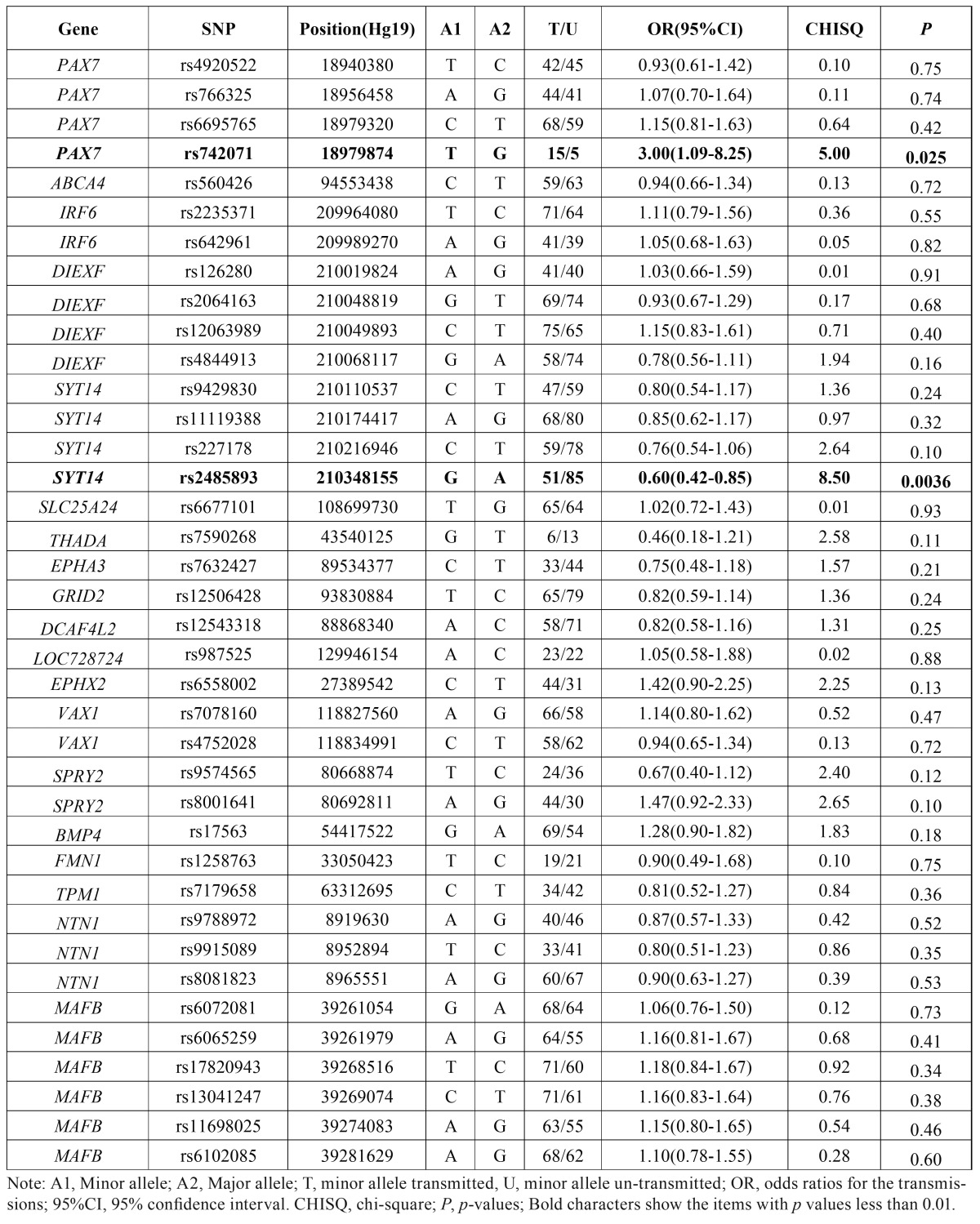
Allelic TDT results of the SNPs for NSCPO.

**Table 3:**
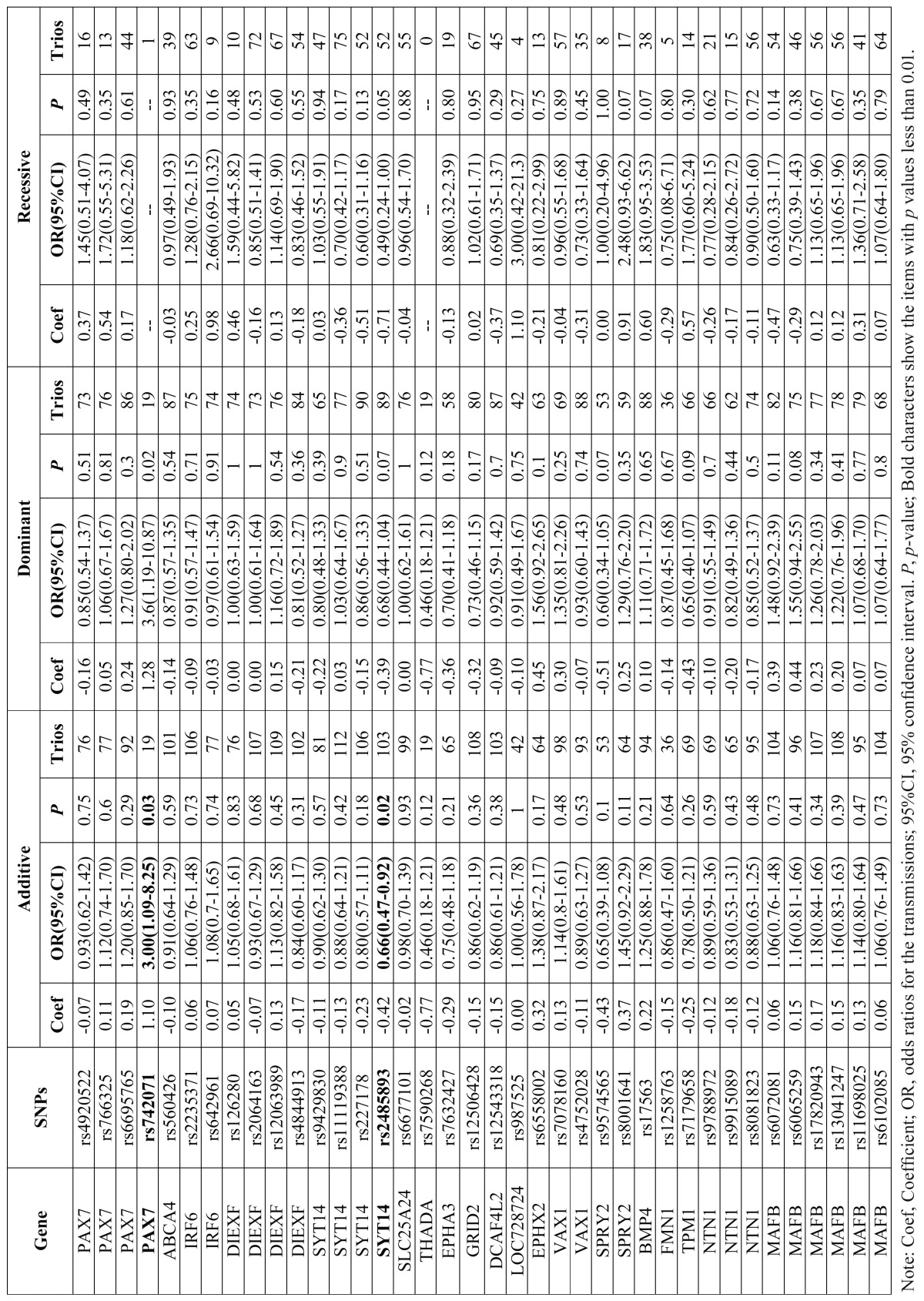
Genotypic TDT Based on 3 Pseudo Controls for NSCPO under different genetic models.

- Parent-of-Origin Effects

There was no significant difference of minor allele transmissions between the maternal and paternal for all SNPs (data not shown). However, we found an excess of maternal transmission of the allele G at rs2485893 (*p*=0.026), allele A at rs8001641 (*p*=0.018) and allele G at rs17563 (*p*=0.045) compared with the paternal ( Table 4), which might warrant future investigations.

**Table 4:**
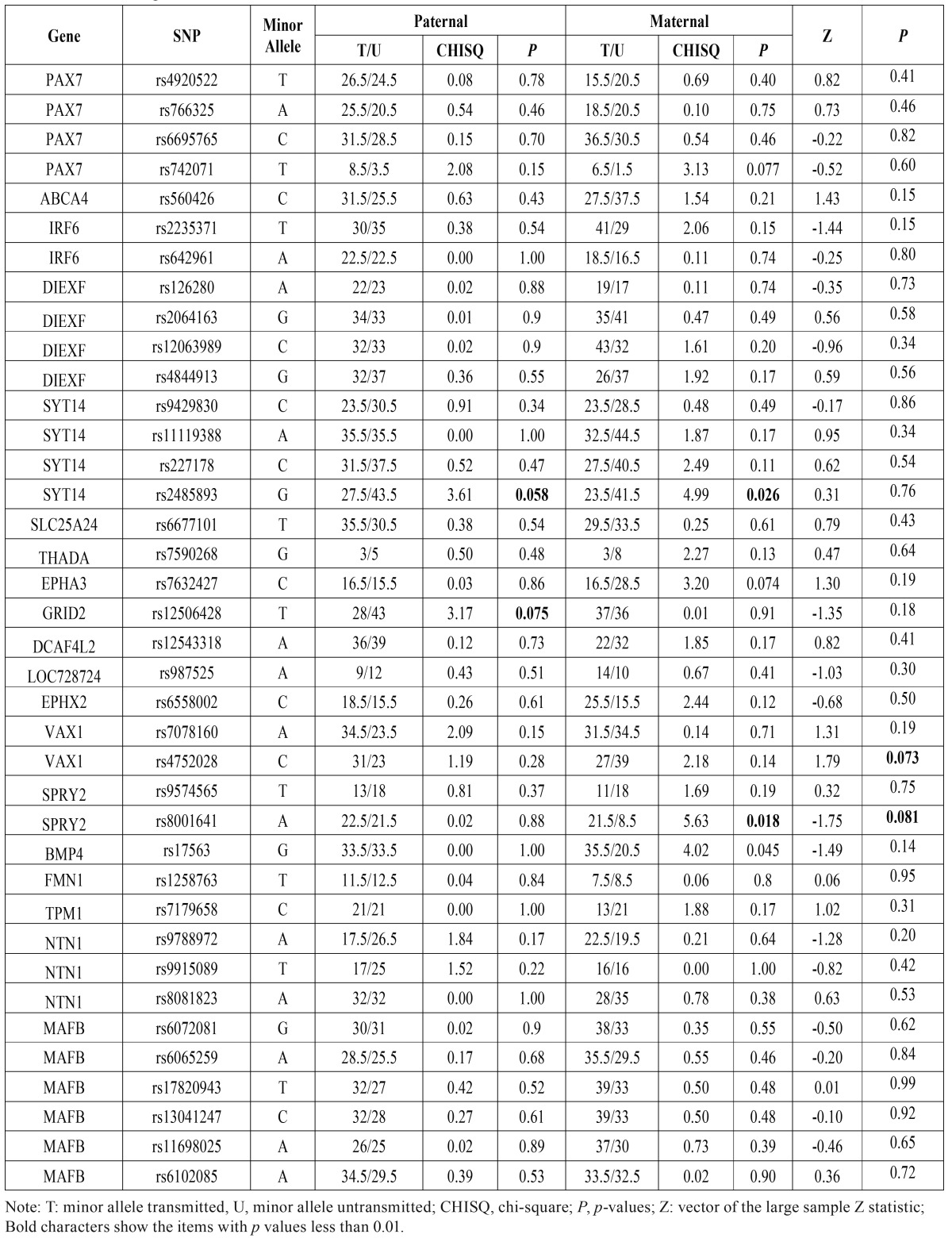
Parent-of-origin effect of effect of the SNPs.

- Gene by Gene Interactions

**Figure 1:**
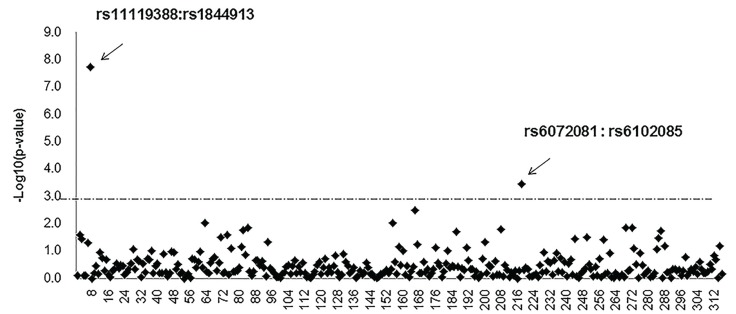
Genotypic TDT for Epistatic Interactions of Pair-wise SNPs.

The Genotypic TDT for epistatic interactions showed that rs4844913 (43kb 3’ of DIEXF) interacted with rs11119388 (SYT14) (*p*=1.80E-08) and rs6072081 (53kb 3’ of MAFB) interacted with rs6102085 (33kb 3’ of MAFB) (*p*= 3.60E-04) for NSCPO (Fig. 1).

- Pair-wise Linkage Disequilibrium and Haplotype Analysis

**Figure 2:**
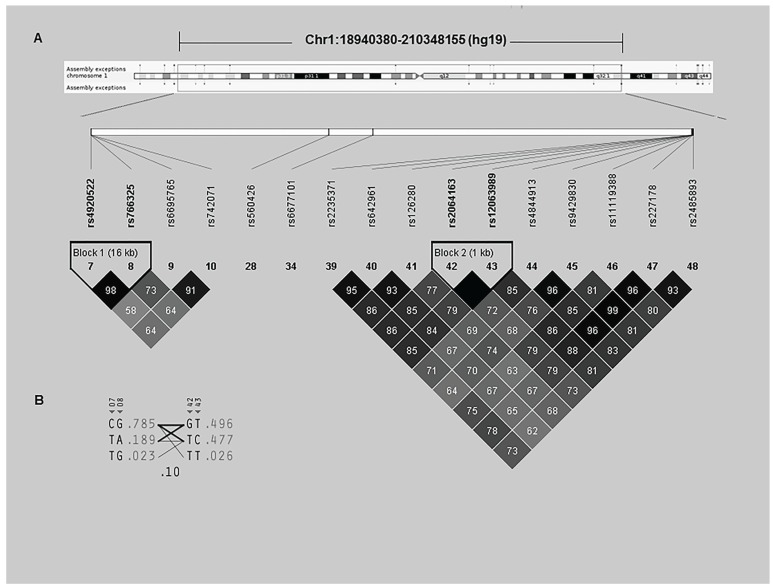
Pair-wise Linkage Disequilibrium (A) and Haplotype (B) of SNPs at Chromosome 1.

We calculated the pair-wise linkage disequilibrium (LD) of SNPs on chromosome 1 based the association results above. There were strong LD between six pairs of SNPs (rs4920552-rs766325, rs126280-rs642961, rs2064163-12063989, rs4844913-rs9429830, rs4844913-rs227178 and rs9429830-227178) with D’>0.93 and r2>0.80, which distributed on two haplotypes (Fig. 2). Based on this, we tried to test if these pairs of SNPs segregate together among NSCPO by carrying out the haplotype analysis. The results did not show any significance (data not shown).

## Discussion

Rs742071 is located in the intron of PAX7 which is involved in neural crest induction and is expressed in cranial neural crest cells, and mice lacking Pax7 have malformations of the nasal and maxillary structures (16). The PAX7 was a second tier GWAS hit (8), later it was confirmed by replication among European and Southeast Asian (17) and GWAS meta-analysis (10). Recently, Leslie *et al.* 2015 performed targeted sequencing of 13 regions from GWASs and other studies in 1,409 Asian and European trios, and carried out a series of statistical and functional analyses, the results indicated that de novo mutation p.Ala259Val disrupted PAX7 function and might contribute to CLP pathogenesis in this individual (18). Although intronic SNPs do not typically alter protein structure, associations with intronic variants have been reported for a number of complex diseases. In this study, we found rs742071 (PAX7) was associated with NSCPO and had a larger genetic effects compared with the associations with NSCL/P from previous GWASs (8,10); Motif analyses by HaploReg indicated that the T allele of rs742071 could greatly alter the affinity of Sin3Ak-20_disc4 (score: 2.1-14.1).

Rs742071 had the minor allele frequency as 0.04 among Han Chinese population, indicating it may need larger sample size to validate its significance; and with the limited sample size, rs742071 did not pass the threshold of the Bonferonni correction p value in the current study. We will add more samples to study it and other variants at PAX7 gene among NSCPO among Han Chinese population.

Rs2485893 was associated with CL/P among Asian ancestry with *p* value 7.86E-07 by GWAS (8). In this study, we found rs2485893 (10kb 3’ of SYT14) was also found to be associated with NSCPO. Marked parent of origin effects were seen for rs2485893 alleles, over-transmission was seen preferentially from mothers compared with fathers ( Table 4). Motif analyses by HaploReg indicated that the G allele of rs2485893 could greatly alter the affinity of AFP1 (score: 2.7-12.3).

Numerous studies have shown that highly conserved non-coding elements act as developmental enhancers *in vivo* (19-21). Non-coding conserved elements around rs742071 and rs2485893 therefore might represent putative regulatory elements for PAX7 and SYT14, we will perform functional studies to elucidate their roles in human craniofacial development.

Gene-gene interactions have been proposed as a potential source of the remaining heritability. Genotypic TDT for epistatic interactions showed that rs4844913 interacts with rs11119388 (SYT14) and rs6072081 interacts with rs6102085 for NSCPO, which provided new insights for the previous GWASs.

In summary, we replicated 38 SNPs contributing to NSCL/P to investigate their roles in NSCPO among Han Chinese population. In this study, we found that rs742071 and rs2485893 were associated NSCPO from Han Chinese population; also, interactions of rs4844913:rs11119388 and rs6072081:rs6102085 for NSCPO were identified, which may provide new insights for the previous GWASs.
